# Long-Term Ocular Outcomes of Prematurity: Morphological Alterations, Visual Aspects and Implications for Age-Related Ocular Diseases

**DOI:** 10.3390/jcm14113667

**Published:** 2025-05-23

**Authors:** Achim Fieß, Sandra Gißler, Eva Mildenberger, Norbert Pfeiffer, Alica Hartmann, Alexander K. Schuster

**Affiliations:** 1 Department of Ophthalmology, University Medical Center Mainz, Johannes Gutenberg University Mainz, 55131 Mainz, Germany; 2 Division of Neonatology, Department of Pediatrics, University Medical Center of the Johannes Gutenberg-University Mainz, 55131 Mainz, Germany

**Keywords:** prematurity, ROP, epidemiology, long-term outcomes

## Abstract

The impact of prematurity has been reported to affect ocular development during infancy and childhood. Research into long-term ocular outcomes in adults born preterm is highly relevant due to a possible impact on the development of age-related ocular diseases such as macular degeneration. The aim was to review the currently available literature regarding outcomes of prematurity on ocular morphology in adults to provide a summary of the long-term effects of prematurity and associated factors such as low birth weight (BW) and retinopathy of prematurity (ROP) and its treatment. Adults formerly born preterm have a higher prevalence of refractive error, lower visual acuity, a higher prevalence of strabismus, shorter axial length, a steeper corneal radius, increased macular thickness, and a thinner peripapillary retinal nerve fiber layer thickness (RNFL), as well as changes in vessel anatomy and the foveal avascular zone. Adults who suffered from ROP have a high risk of myopic refractive error, amblyopia, shallower anterior chambers and thicker crystalline lenses, higher corneal aberrations, thinner RNFL thickness, and foveal hypoplasia. Individuals with advanced ROP requiring treatment also have higher rates of astigmatism, an increased temporal RNFL thickness, altered macular curvature, and reduced visual acuity. Prematurity leads to lifelong ocular morphological and functional changes, suggesting that fetal origins may contribute to age-related ocular diseases. This could have implications for ophthalmologic monitoring and the frequency of check-ups in adulthood.

## 1. Introduction

An estimated 13.4 million babies were born before term (<37 weeks) in 2020, with a global prevalence of preterm birth of 9.9% [1]. The burden of preterm birth impacts the health and lives of these children and their families, leading to an increased risk of lifelong cardiovascular, metabolic, respiratory, kidney, psychiatric, and neurodevelopmental disorders [2]. These complications contribute to higher general morbidity and mortality of those born preterm up to late adulthood [3].

Ophthalmic research has contributed to many discoveries regarding the outcome of preterm birth on ocular morphology from infancy to adolescence, ranging from reduced visual acuity [4,5] to multiple changes in the anterior [6,7,8] and posterior eye segment [9,10,11,12,13,14,15,16]. Low gestational age (GA) is a risk factor for the development of strabismus, amblyopia, and refractive errors during infancy and childhood [17,18,19]. Structural alterations to the optic radiations may contribute to the impaired vision of those born preterm [20], which is significantly related to lower educational attainment and economic potential in adolescence [21].

Although several smaller studies have explored the ocular outcomes of prematurity into adulthood, four major studies have been conducted to date. Darlow et al. investigated the eye function and morphology of individuals born preterm (n = 250; birth weight (BW) ≤ 1500 g, mean GA 29.2 ± 2.6) aged between 27 and 29 years who underwent screening for retinopathy of prematurity (ROP) after birth; however, no treatment was administered at this time as standard protocols had not yet been established [4,22,23]. Pétursdóttir et al. examined the visual function and eye morphology of 59 individuals born preterm (BW ≤ 1500 g, mean GA 29.3 ± 2.1) compared to 44 individuals born full-term, aged between 25 and 29 years [24,25,26,27,28]. The German Gutenberg Health Study (GHS) compared several morphological parameters in adult individuals across different birth weights (low BW < 2500 g, n = 458; normal BW 2500–4000 g, n = 6854; high BW > 4000 g, n = 1057). However, the lack of data on ROP status and GA in this cohort limits the interpretation of their findings regarding prematurity [29,30,31,32,33,34,35,36,37,38,39,40,41,42,43]. The Gutenberg Prematurity Eye Study (GPES; n = 450) has further advanced the understanding of the long-term outcomes of prematurity and included comparisons of subjects with low GA, a low BW percentile, ROP and ROP treatment later in life. Participants included 140 term-born individuals (Group 1), individuals born moderately preterm (gestational age 33–36 weeks (wks), n = 137, Group 2), individuals born very preterm (GA 29–32 wks, n = 92, Group 3), individuals born extremely preterm (GA ≤ 28 wks, n = 18, Group 4), individuals who developed ROP (n = 48, Group 5) and individuals who underwent ROP treatment (n = 15, Group 6, laser-treatment: n = 7; cryocoagulation: n = 8) aged between 18 and 52 years [29,32,40,44,45,46,47,48,49,50,51,52,53,54,55,56,57,58,59].

Morphological eye alterations persisting into adulthood may predispose individuals to the development of age-related diseases such as age-related macular degeneration and glaucoma. This narrative review examines the impact of prematurity and associated factors on ocular morphology in adulthood, with a particular focus on potential links to age-related eye diseases. Although the long-term ocular effects of prematurity are increasingly recognized, the current literature lacks a comprehensive synthesis that clearly differentiates the individual contributions of low gestational age, low birth weight, and retinopathy of prematurity (ROP), including its treatment, to adult ocular outcomes. Distinguishing these factors is essential, as they are interrelated yet may contribute uniquely to long-term ocular outcomes. A clearer understanding of their individual roles is essential for refining clinical interventions and improving long-term prognoses. To provide a comprehensive perspective, the review primarily focuses on the four major studies in the field while incorporating results from several smaller investigations. While the primary emphasis is on adult outcomes, relevant pediatric studies are included where adult data are limited or where treatment is only possible in younger children (e.g., ROP treatment, amblyopia). The goal is to summarize the current knowledge, refine clinical understandings of the issue, and highlight future research directions aimed at improving long-term ocular health in individuals born preterm.

## 2. Visual Acuity

In the two-country birth cohort study, adults born with very low birth weight (VLBW) exhibited lower best corrected visual acuity (BCVA), reduced contrast sensitivity thresholds, and lower scores on self-reported vision-targeted health status compared to controls [60]. However, ROP status was not included in this assessment, which is an important limitation. This omission is relevant because another study demonstrated that adults with VLBW who developed ROP had reduced visual acuity compared to adults with no ROP and controls. Furthermore, the individuals drove cars less frequently and reported greater difficulties with daily activities due to their eyesight [22]. A longitudinal study additionally found that visual acuity, though reduced, remained stable over a 20-year follow-up period [4]. Smith et al. examined participants aged 45 to 56 years who had experienced untreated ROP and found that visual acuity was 20/200 or worse in more than 50% of the eyes [61].

With regard to the specific impact of prematurity rather than low BW, individuals born preterm demonstrated significantly lower distance visual acuity (DCVA), near visual acuity, and contrast sensitivity compared to those born full-term, even after excluding individuals with previous ROP or neurological complications. Advanced ROP requiring treatment has been identified as a risk factor for reduced near visual acuity and sensitivity of the central visual fields [25]. BCVA among eyes of individuals born extremely preterm was significantly worse, with the greatest impairment seen in groups with advanced stages of ROP requiring treatment and even among those with untreated ROP [62]. In the GPES, lower DCVA was associated with lower GA, lower BW, the presence of ROP, ROP treatment, and perinatal adverse events. Vision-related quality of life (VRQoL) scores were lower among individuals born preterm regardless of their ROP status, although socioemotional VRQoL scores were lower only in individuals with advanced stages of ROP requiring treatment [44]. Health-related quality of life (HRQoL) was also significantly lower in individuals born preterm if they had visual impairments and additional impairments [63], which are also more common in individuals born preterm, as discussed in the following paragraphs. These findings highlight the lasting impact of perinatal parameters on visual functioning into adulthood. However, good visual outcomes can still be achieved in individuals with regressed ROP if regular follow-up examinations and prompt treatments for any amblyogenic conditions are provided [64].

## 3. Strabismus and Amblyopia

**Figure 1:**
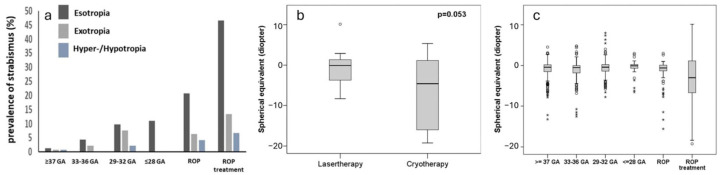
Strabismus and spherical equivalent in the Gutenberg Prematurity Eye Study; (**a**) prevalence of esotropia and hypermetropia/hypometropia in the Gutenberg Prematurity Eye Study, Reprinted with permission from Fieß et al., British Journal of Ophthalmology, published by BMJ Publishing Group, 2024 [32], (**b**) type of ROP treatment in the different groups in the Gutenberg Prematurity Eye Study, Reprinted with permission from Fieß et al., Acta Ophthalmologica, published by John Wiley and Sons, 2022 [49], (**c**) descriptive results regarding spherical equivalent, Reprinted with permission from Fieß et al., Acta Ophthalmologica, published by John Wiley and Sons, 2022 [49]; GA—gestational age (weeks), ROP—retinopathy of prematurity; *—extreme outlier (Figure 1c).

Strabismus and amblyopia contribute to reduced visual acuity, impaired or absent stereopsis, and decreased VRQoL [65]. Significantly higher prevalence of strabismus [26], nystagmus, and abnormal ocular motility was reported in individuals born extremely preterm, and these associations are at least partially independent of ROP status [62]. Esotropia (but not exotropia) and anisometropia have been specifically linked to lower GA [66]. Figure 1a shows that the prevalence of strabismus is highest in adults with advanced ROP requiring treatment (60%), followed by those with untreated ROP (27.1%) and those born with a GA of 33–36 weeks (17.4%) compared to only 2.1% in full-terms controls. In the multivariable analyses, strabismus was associated with GA, anisometropia, hypermetropia and astigmatism, with esotropia occurring more frequently than exotropia [32].

Refractive errors and strabismus during early childhood pose an additional risk for the development of amblyopia. Likewise, specific perinatal factors in children born preterm are related to amblyopia development [67]. In the GPES, amblyopia prevalence was increased in adults with a history of ROP [44]. As amblyopia nearly doubles the lifetime risk of affected individuals to suffer from bilateral vision impairment [68] and treatment becomes markedly less effective beyond mid-childhood, systematic orthoptic screening, prompt refractive correction, and thorough longitudinal follow-up are essential for infants and younger children born preterm. Early detection and intervention not only reduce the incidence of amblyopia but also preserve stereopsis and optimize VRQoL throughout life.

## 4. Refractive Error

Darlow et al. found that high myopia (>−5 diopters (D)) was more frequent in adults born with VLBW who had a history of ROP, compared to those without and to controls [22]. While several studies have reported an association between birth weight and refractive error in adulthood, many did not include information on ROP status, limiting the interpretability of their findings [39,69]. Axial length and lenticular changes appear to be the primary contributors to these observed differences in spherical equivalent in adulthood [31,70].

Regarding the impact of GA, Pétursdóttir et al. [27] found that individuals born preterm were more likely to be either myopic or hyperopic and had a higher prevalence and degree of astigmatism and anisometropia. The risk of myopia and astigmatism tended to be highest in individuals with advanced ROP requiring treatment, although these findings did not reach statistical significance [27]. The GPES reported that only participants with advanced ROP requiring treatment had an increased refractive error, whereas individuals born preterm without ROP—or with ROP that did not require treatment—did not show increased refractive error, which is clearly displayed in Figure 1c [49]. As displayed in Figure 1b, descriptively, the myopic refractive error was more pronounced in participants treated with cryocoagulation compared to those who underwent laser therapy [49]. This represents an important contribution of the study as, to our knowledge, this is the only study that has compared the outcome of types of ROP treatment in adulthood. Furthermore, only GA was associated with anisometropia and anisoastigmatism in adulthood [49]. Accommodation amplitude was also significantly reduced in eyes with advanced stages of ROP requiring treatment, relative to the control group [49]. Although newer treatment modalities, such as anti-VEGF injections, have shown smaller effects on refractive error in childhood, long-term data into adulthood are currently lacking [71,72]. Refractive errors in individuals born preterm without ROP are mainly attributed to variations in axial length. In contrast, in those with ROP, lenticular changes may also play a significant role in the development of myopia [70].

Longitudinal follow-up studies have shown both increasing and decreasing alterations in refractive error from adolescence into young adulthood [22,27]. However, differing inclusion criteria—one study focused on low BW and the other on prematurity—might explain the conflicting results. Further follow-up studies are still needed, especially in older adulthood, to better understand the long-term effects of prematurity and its associated factors on refractive error development.

## 5. Anterior Segment of the Eye: Ocular Geometry

Results from the GHS suggest that, in adulthood, lower BW is associated with decreased horizontal trefoil, higher spherical aberrations, increased higher-order aberrations, and increased lower-order aberrations [42]. Lower BW was also linked to thinner corneal thickness [43], steeper corneal curvature, a smaller white-to-white distance, and shorter axial length [40]. In contrast, the anterior chamber depth and lens thickness were not associated with low BW [40]. According to the GHS, axial length variation may explain the majority of variance in the refractive error observed in adulthood [31].

**Figure 2:**
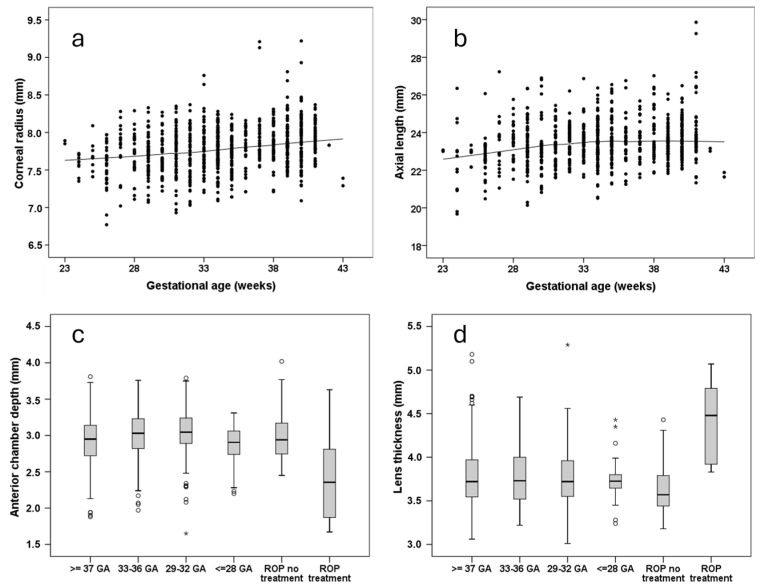
Anterior findings in the Gutenberg Prematurity Eye Study; (**a**) corneal radius, (**b**) axial length, (**c**) anterior chamber depth, and (**d**) lens thickness in the Gutenberg Prematurity Eye Study; all reprinted with permission from Fieß et al., British Journal of Ophthalmology, published by BMJ Publishing Group, 2024 [52]; GA—gestational age (weeks), ROP—retinopathy of prematurity; *—extreme outlier.

A steeper corneal radius was also associated with lower GA (Figure 2a) and a lower BW percentile, whereas shorter axial length was only associated with lower GA (Figure 2b) [52]. ROP treatment was linked to shallower anterior chamber depth (Figure 2c), a smaller anterior chamber angle [ACA] width [59], and increased lens thickness (Figure 2d) [52], which may predispose individuals to acute angle-closure glaucoma in later life. Multivariable analyses further demonstrated an association of a lower BW percentile with decreased corneal thickness at the apex. This finding suggests that restricted intrauterine growth, rather than low BW per se, affects the corneal thickness, while neither GA, ROP occurrence, nor ROP treatment showed an association [55].

Regarding corneal aberrations, the GPES identified associations between higher-order aberrations and both a lower BW percentile and advanced stages of ROP requiring treatment [47]. As the GHS only assessed BW, it is reasonable to assume that intrauterine growth restriction, rather than low BW, is the main factor affecting corneal surface morphology. Corneal lower-order aberrations were also associated with a lower BW percentile and advanced stages of ROP requiring treatment, but not with untreated ROP [47]. Since increased corneal aberrations correlated with lower visual acuity and greater refractive error in the GPES [47], factors regarding preterm birth appear to be highly relevant for long-term visual outcomes into adulthood. Moreover, morphological differences in the anterior segment may not only contribute to reduced visual acuity but also increase the risk of developing age-related diseases such as cataracts [73] or glaucoma [74].

## 6. Posterior Segment of the Eye

### 6.1. Macula and Fovea

The development of the posterior pole is crucial for the adequate processing of visual information. The development of the foveal pit begins to form around 22 weeks postmenstrual age and the fovea reaches maturity by approximately 45 months of age [75]. It is suspected that the abrupt environmental changes associated with preterm birth may disrupt foveal development.

In adulthood, participants with a history of ROP exhibited increased macular thickness and persistence of inner retinal layers within the fovea [76]. Reduced foveal depth was also found in individuals born preterm who had undergone cryotherapy for ROP [77], and mean foveal thickness was reported to be negatively associated with the severity of ROP stage [78]. Among eyes in individuals born extremely preterm, both inner and outer retinal layers were thicker, especially in eyes treated with laser or cryotherapy. Eyes with thicker retinal layers also had a lower BCVA [79]. However, even independent of ROP, GA seems to be a risk factor for increased macular thickness [28].

Furthermore, low BW was also associated with increased retinal thickness and a higher prevalence of foveal hypoplasia [41]. One study assessing VLBW in adults born preterm observed thicker retinal layers in the foveal area associated with GA. Contrary to earlier studies, this did not affect the BCVA [80]. It should be noted that the ROP status of participants was not evaluated in that study, limiting the interpretation of the results.

**Figure 3:**
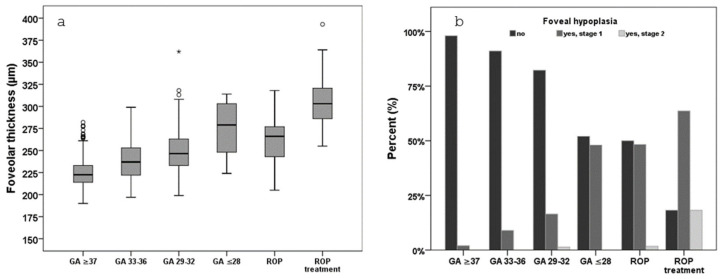
Retinal thickness and foveal hypoplasia in the Gutenberg Prematurity Eye Study; (**a**) foveolar thickness in all participant groups in the GPES, (**b**) foveal hypoplasia stages in all participant groups in the GPES, adapted from [48]; GA—gestational age (weeks), ROP—retinopathy of prematurity, GPES—Gutenberg Prematurity Study; *—extreme outlier.

Findings in adults show that central foveal thickness and the prevalence of foveal hypoplasia increased with decreasing GA, with individuals with ROP (with or without treatment) (Figure 3a) demonstrating the highest rates of foveal hypoplasia. Figure 3b additionally provides the reader with the stages of foveal hypoplasia distributed over the groups of GA and ROP, of which higher stages were mainly found in adults who were treated for ROP (Figure 3b). In this study, visual acuity was lower in those individuals with increased foveolar thickness and foveal hypoplasia [48]. Furthermore, a sex-specific correlation between lower GA and thicker central foveal thickness was identified, demonstrating that GA had a more pronounced effect on central foveal thickness in male participants [46]. More recent results suggest that hypotrophy does not affect foveal retinal thickness, implying that only prematurity and its associated factors, other than fetal growth restriction, are responsible for this developmental disorder [53].

Additionally, the GPES found that advanced stages of ROP requiring treatment and maternal smoking were associated with altered macular curvature. Adults with advanced stages of ROP requiring treatment had a higher prevalence of negative macular curvature values compared to full-term controls, indicating a more pronounced macular protrusion toward the vitreous cavity, which could negatively affect visual acuity in adulthood [54]. In the UK Biobank study, a negative association between BW and macular curvature was found, as well as a lower curvature with maternal smoking [81].

### 6.2. Retinal Nerve Fiber Layer and Optic Disc

**Figure 4:**
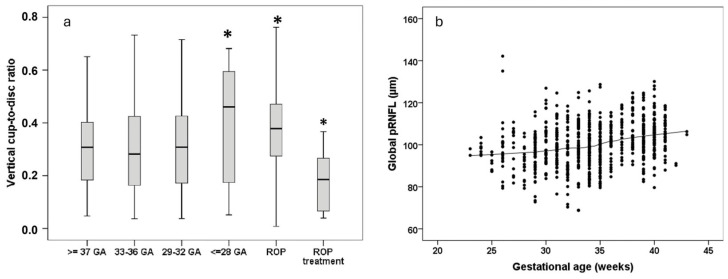
Vertical cup-to-disc ratio and global peripapillary retinal nerve fiber layer thickness in the different participant groups in the Gutenberg Prematurity Eye Study; (**a**) vertical cup-to-disc ratio in all participant groups in the Gutenberg Prematurity Eye Study, reprinted with permission from Fieß et al., American Journal of Ophthalmology, published by Elsevier, 2022 [51], (**b**) global peripheral retinal nerve fiber layer thickness distributed over gestational age, reprinted with permission from Fieß et al., American Journal of Ophthalmology, published by Elsevier, 2022 [50]; GA—gestational age, ROP—retinopathy of prematurity; *—*p* < 0.05.

The peripapillary retinal nerve fiber layer (pRNFL) bundles the visual information into the optic nerve, allowing transmission to the brain for neurological processing. In adulthood, participants who underwent cryotherapy for ROP had an increased thickness of the temporal pRNFL quadrant, particularly those with pronounced retinal dragging [77]. Additionally, global pRNFL thickness is reduced in individuals born preterm, of which the descriptive distribution is displayed in Figure 4b, although this reduction is not associated with the occurrence of ROP [50]. An analysis of individuals born full-term found that extreme hypotrophy is an additional risk factor for thinner pRNFL [82]. Pétursdóttir et al. [28] also observed that the average pRNFL thickness was lower in individuals born preterm, even after excluding participants with prior ROP and/or neurological complications. Conversely, advanced stages of ROP requiring treatment were correlated with thicker average RNFL [28]. The GHS also confirmed an association between BW and pRNFL thickness [83], while the two-country birth cohort study found moderate thinning of the neuroretinal rim and pRNFL in adults born preterm with VLBW [84].

Changes in the optic disc, particularly in the vertical cup-to-disc ratio (VCDR), can also be seen in individuals born preterm. In adulthood, VCDR was significantly larger in individuals born at a GA ≤ 28 weeks without ROP and in individuals with untreated ROP, whereas participants with advanced stages of ROP requiring treatment exhibited a smaller VCDR compared to full-term controls (Figure 4a) [51]. These results are in line with observations in infancy [85] and align with the pattern of thinner pRNFL in adults born preterm and thicker pRNFL in adults who received treatment for ROP [50]. A low BW percentile has also been identified as risk factor for thinner pRNFL and larger VCDR, suggesting an impact of hypotrophy even in the absence of prematurity [82]. However, BW was not associated with the prevalence of glaucoma and VCDR on a population-based level [37]. The leading theory behind alterations in pRNFL thickness is transsynaptic degeneration, meaning that effects related to prematurity affect brain development leading to further degeneration of optic nerve fibers [86]. This could lead to a thinner pRNFL and a larger VCDR, potentially resulting in the reduced reserve capacity of optic nerve fibers, which might become clinically relevant in conditions such as glaucoma. Additional long-term studies are needed in older cohorts to follow up on potential decreases in nerve fiber layers among adults born preterm.

### 6.3. Retinal Vasculature and Choroid

Lower GA has been associated with a greater tortuosity of retinal arteries in adulthood, although this alteration does not appear to impact visual acuity [87]. It has been hypothesized that preterm birth could result in a lower threshold for the development of vascular disease [88], potentially due to the combination of abnormal retinal vascularization and elevated blood pressure observed in adult women born preterm. In the same study, the length index for arterioles was higher and there were fewer vessel branching points [88]. Other studies found an association between lower BW and a narrower arteriolar caliber, although the venular caliber appeared unaffected [89]. However, both the GPES and the GHS found no significant differences between adults with low BW or GA and controls [36,45]. Therefore, the evidence regarding the caliber of the vessels remains conflicting.

Regarding the choroid, the mean choroidal thickness was found to be significantly thinner in young adults with regressed ROP, with no significant correlation with GA, BW, or visual acuity [13]. The GPES found that a low BW percentile was associated with a thinner peripapillary choroidal thickness [57], yet this alteration did not seem to affect visual acuity [57].

**Figure 5:**
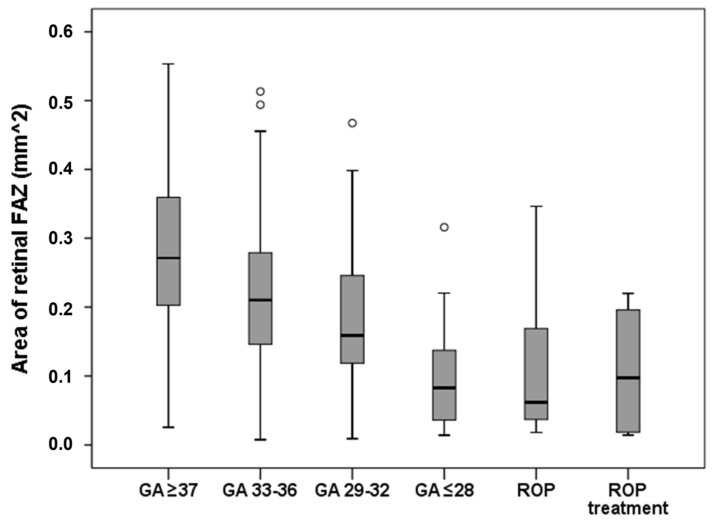
Foveal avascular zone in the Gutenberg Prematurity Eye Study. Area of the foveal avascular zone in the different participant groups in the Gutenberg Prematurity Eye Study, adapted from [56]; GA—gestational age (weeks), ROP—retinopathy of prematurity, FAZ—foveal avascular zone.

Another important factor in retinal vasculature development is the size of the foveal avascular zone (FAZ). The FAZ precedes the formation of the foveal pit [90] and may be related to the development of it [91]. Furthermore, a smaller FAZ is associated with a narrower foveal pit opening and a thicker fovea [90]. A lower GA is associated with a smaller FAZ (Figure 5) and a smaller FAZ is, in turn, linked to a higher prevalence of foveal hypoplasia, although this is not directly associated with reduced visual acuity [56]. Preterm birth has also been associated with decreased plexus vessel density in both the superficial and deep capillary plexus [92]. Importantly, alterations in FAZ size may play a role in the development of age-related macular degeneration (AMD) and/or diabetic retinopathy [93], a relationship that warrants further investigation.

## 7. Ocular Diseases with Fetal Origins in Adulthood

Data regarding the effects of prematurity and associated factors in terms of age-related diseases are scarce, as many studies focus on the outcomes of individuals with ROP. As there have been cases of acute angle-closure glaucoma associated with ROP [94], we recommend that researchers conduct long-term follow-up studies of individuals formerly born preterm with ROP. Furthermore, ocular surgeries result in more complications in patients with a history of premature birth and ROP [95,96], e.g., phthisis bulbi [97]; in these individuals, cataract surgery tends to be performed at a younger age [96].

The impact of GA and low BW might both be of importance regarding the effects on ocular morphology discussed previously. An association of refractive error with AMD has been described [98] and, as adults born preterm are prone to more severe refractive abnormalities and cardiovascular risk factors [99], they might also have an increased risk of developing AMD. Furthermore, a link between overall AMD prevalence and low BW has been reported in the GHS, although these results were limited by missing data and loss to follow up [34]. Additionally, the GHS discovered that diabetic retinopathy was associated with high and low BW mediated by diabetes duration and arterial hypertension [38]. While associations between prematurity and age-related ocular diseases such as AMD have been suggested, the current evidence remains limited; therefore, these links are presented as hypotheses rather than established outcomes.

## 8. Critical Appraisal of the Literature

When reviewing the available evidence, several important limitations must be acknowledged. Many studies investigating long-term ocular outcomes following prematurity are constrained by small sample sizes, limiting their statistical power and generalizability. Cohort heterogeneity is also a significant concern, as populations often vary in terms of the degree of prematurity, neonatal care practices, and the severity of early ophthalmologic disease. Additionally, treatment era effects must be considered: advances in neonatal care and the introduction of new therapies, particularly anti-VEGF agents for ROP, have substantially changed clinical outcomes, making it difficult to extrapolate findings from older cohorts to individuals treated with contemporary approaches. Another major limitation is the lack of adjustment for potential confounding factors, such as socioeconomic status, neurodevelopmental disorders, and systemic health conditions, all of which may independently influence ocular health outcomes. The omission of these factors weakens the strength of reported associations between prematurity and ocular disease in adulthood. Together, these limitations highlight the need for the cautious interpretation of the current data and underscore the importance of future longitudinal studies with larger, more homogeneous, and contemporarily treated cohorts that account for key confounders.

In summary, this study provides an overview of several aspects of ocular morphology in adults born preterm or with low BW (Supplemental Table S1), showing that the variety of ocular morphological effects of prematurity is reflected in all parts of the eye and persists until adulthood. Further follow-up studies of older participants are necessary to gain additional knowledge regarding age-related diseases, in order to estimate the impact of prematurity on both the prevalence and incidence of these diseases. Additionally, there needs to be an emphasis on a comparative assessment of the different effects of available treatment types of ROP on ocular morphology in early and late adulthood. Understanding the impact of prematurity-related factors will enable healthcare providers to implement targeted screening and intervention strategies to improve visual outcomes and reduce the long-term impact on individuals’ visual development.

## Supplementary Materials

The following supporting information can be downloaded at: https://www.mdpi.com/article/10.3390/jcm14113667/s1, Table S1: Overview of effects of prematurity and its associated factors on ocular morphology in adulthood.

## Abbreviations

The following abbreviations are used in this manuscript:

GA	Gestational age
BW	Birth weight
ROP	Retinopathy of prematurity
GHS	Gutenberg Health Study
GPES	Gutenberg Prematurity Eye Study
VLBW	Very low birth weight
BCVA	Best-corrected visual acuity
DCVA	Distant-corrected visual acuity
VRQoL	Vision-related quality of life
D	Diopter
VEGF	Vascular endothelial growth factor
ACA	Anterior chamber angle
pRNFL	Peripapillary Retinal Nerve Fiber Layer
VCDR	Vertical cup-to-disc-ratio
FAZ	Foveal avascular zone
AMD	Age-related macular degeneration
