# Supplementary material for: Long-Term Ocular Outcomes of Prematurity: Morphological Alterations, Visual Aspects and Implications for Age-Related Ocular Diseases
Source: J Clin Med. 2025 May 23;14(11):3667. doi: 10.3390/jcm14113667 (PMC12155540; doi:10.3390/jcm14113667)
Supplement: Supplementary file 1 [file jcm-14-03667-s001.zip › jcm-3557565-supplementary.pdf]

Table S1.

Overview of effects of prematurity and is associated factors on ocular morphology in adulthood

**Gutenberg Prematurity Eye Study (GPES)<br>(n=450, aged 18-52 years)**		
**Preterm birth (Low GA)**	Refractive Error	no difference in spherical equivalent of low GA compared to full-terms [1]
	Strabismus	risk factor for strabismus [2]
	Anterior segment	steeper corneal radius, smaller white-to-white distance [3]
		increased bulbar redness, increased length of wetting in the Schirmer test, larger narrowing of the nasal palpebral angle [4]
		no effect on corneal thickness [5]
		unaltered ACA width [6]
	Axial Length	decreased axial length [3]
	Macula & Fovea	central foveal thickness and foveal hypoplasia was independently associated with lower GA [7]

		smaller FAZ [8]
	RNFL	global pRNFL thinning [9]
	Optic Disc Morphology	larger VCDR [10]
**Low BW Percentile**	Anterior segment	steeper corneal radius [3]
		smaller white-to-white distance [3]
		association with lower corneal thickness at the apex & the pupil [5]
		association with corneal higher-order & lower-order aberrations [11]
	RNFL	no association with global pRNFL thinning [9]
**ROP**	Amblyopia	higher frequency of amblyopia [12]
	Anterior segment	smaller white-to-white distance [3]
		no effect on corneal thickness [5]

		unaltered ACA width [6]
		corneal lower-order aberrations were NOT associated [11]
	RNFL	global pRNFL thinning was NOT associated with ROP occurrence [9]
	Optic Disc Morphology	larger VCDR [10]
**ROP-treatment**	Visual Acuity	decreased visual acuity [12]
	Refractive Error	lower accommodation [1]
	Anterior segment	more lens opacifications [1]
		shallower anterior chamber depth, increased lens thickness [3]
		lower F-NITBUT (<20 s) [4]
		no effect on corneal thickness [5]
		smaller anterior chamber angle width in nasal & temporal positions [6]

		corneal higher-order aberrations & lower BW percentile [11]
	Macula & Fovea	association with macular curvature [13]
		association with central foveal thickness [9]
	RNFL	increased pRNFL in the temporal sector [7]
	Optic Disc Morphology	smaller VCDR, more likely to have a torted disc [10]
**Gutenberg Health Study (GHS) (n=15 010, aged 35-74 years at Baseline)**		
**Low BW**	Visual Acuity	association with spherical equivalent & BCVA, high BW: association with spherical equivalent, none with BCVA [14]
	Anterior segment	association with decreased horizontal trefoil, higher RMS, increased HOA & increased LOA [15]
		association with thinner corneal thickness at the apex and the pupil, effects diminished towards the corneal periphery, no differences in the perilimbal regions [16]
		association with steeper corneal curvature, smaller white-to-white distance, thinner central corneal thickness, & shorter axial length. No association with anterior chamber depth & lens thickness [17]
	RNFL	association with increased foveolar thickness & foveal hypoplasia [18]

	Optic Disc Morphology	BW: association with pRNFL thickness in the global sector [19]
	Ocular diseases	low and high BW in diabetics: higher risk of diabetic retinopathy [20]
		higher prevalence of total AMD, in particular early AMD prevalence [21]
**Pétursdóttir et al. (n=103, aged 25-29 years)**		
**Preterm birth (Low GA) (mean GA 29.3±2.1 weeks)**	Visual Acuity	lower distance visual acuity, near visual acuity, mean deviation & contrast sensitivity [22]
	Refractive Error	higher prevalence of anisometropia and astigmatism [23]
	Strabismus	higher prevalence of strabismus, reduced stereoacuity & worse amplitude of accommodation [24]
	Anterior segment	corneal radius was shorter in preterms, AL/CR ratio was similar in preterms and full-terms [23]
	Axial length	axial length was shorter in preterms, AL/CR ratio was similar in preterms and full-terms [23]
	Macula & Fovea	increased central macular thickness was increased, thinner macular GC-IPL [25]
		thicker macula but no reduced GC-IPL [25]

	RNFL	thinner average peripapillary RNFL [25]
**ROP-treatment**	Anterior segment	cryotherapy was related with astigmatism [23]
**Further studies regarding preterm birth**		
**Preterm birth (Low GA)**	Visual Acuity	worse mean (SD) BCVA [26]
	Refractive Error	higher prevalence of SE ≥ 1.5 D, anisometropia ≥ 1.0 D & astigmatism ≥ 1.0 D [27]
	Strabismus	higher prevalence of strabismus, abnormal ocular motility & nystagmus [26]
		association with esotropia [28]
	Anterior segment	shorter CR, AL/CR ratio was similar to term-birth [27]
	Axial Length	shorter CR, AL/CR ratio was similar to term-birth [27]
	Macula & Fovea	thicker inner & outer retinal layers, reduced BCVA [29]
	Vessels /Choroid	women born preterm: higher length index for arterioles & significantly fewer number of vascular branching points [30]

Further studies regarding ROP		
**ROP**	Visual Acuity	VLBW with ROP: reduced visual acuity, 20-year-FU: stable rates of visual acuity but increased myopia [31]
	Refractive Error	VLBW with ROP: more high myopia (>5 D) [31]
	Anterior segment	significantly shorter axial lengths, shallower anterior chambers, & thicker lenses [32]
		LT was significantly larger & ACD significantly shorter in 360 degrees - ROP group than in partial group, no differences between 360 & partial in SER, AL & CCR [33]
	Macula & Fovea	negative correlation between ROP stage & MFT, association with Ellipsoid zone (EZ) abnormalities, inner retinal layer thickening, & presence of chorioretinal atrophy [34]
		relative loss of the foveal depression, increased macular thickness, & continuation of inner retinal layers within the fovea [35]
	Ocular diseases	late-onset exudation & fibrovascular proliferation occur rarely with previously regressed ROP [36]
		acute angle-closure glaucoma developed in three adults who were being followed up for stable cicatricial ROP [37]
		repair of a tear or detachment in such a patient is more likely to require multiple procedures but can still be associated with good visual results [38]
		cataract surgery tends to be performed at a young age, has a mixed range of visual results, & can be associated with a high rate of retinal complication [39]

		high risk of vision-threatening complications throughout childhood & adulthood [40]
		various late retinal findings & complications, especially retinal detachments, complications in all age groups [41]
	Miscellaneous	VLBW adults with ROP drove a car less often & had higher difficulties with everyday activities scores due to eyesight [31]
**Further studies regarding ROP-treatment**		
**ROP-treatment**	Visual Acuity	decreased vision [29]
		reduced near visual acuity & visual fields [22]
	Refractive Error	cryotherapy: correlation with astigmatism, not with SE & anisometropia [27]
	Macula & Fovea	thicker macula [25]
		reduced foveal depth [42]
		increased inner & outer retinal layer thickness [29]
	RNFL	increased average RNFL [25]

		increased thickness of the temporal retinal nerve fiber layer, especially when pronounced retinal dragging [42]
	Miscellaneous	more problems resulting from cerebral dysfunction [43]
**Further studies regarding low birth weight**		
**Low BW**	Visual Acuity	VLBW with ROP: reduced visual acuity compared with no ROP & controls. 20-year FU: rates of reduced visual acuity stable, myopia increased [31]
		VLBW: lower BCVA ETDRS score & lower contrast sensitivity thresholds [44]
		VLBW: lower contrast sensitivity [45]
	Refractive Error	little to no role in the development of myopia [46]
		more myopic refractive error (only modest causality impact) [27]
	RNFL	thicker retinal nerve fibre layer [45]
	Miscellaneous	VLBW with ROP: drive a car less often, higher difficulties with everyday activities due to eyesight [31]

Abbreviations: GA - Gestational age; ACA – Anterior chamber angle; FAZ – Foveal avascular Zone; (p)RNFL – (peripheral) retinal nerve fibre layer; (V)CDR – (Vertical) Cup-to-disc-ratio; (VL)BW – (very low) birth weight; F-NITBUT – First Non-invasive tear break-up-time; BCVA – best corrected visual acuity; DCVA – distant corrected visual acuity; EDTRS - Early Treatment Diabetic Retinopathy Study; ROP – retinopathy of prematurity; SE(R) – spherical equivalent (refraction); MFT – Mean foveal thickness; RMS – root mean square; HOA – higher order aberrations; LOA – lower order aberrations; FU – Follow-up; CR – corneal radius; CCR – corneal curvature radius; AL/CR – axial-length/corneal-radius-ratio; LT – lens thickness; ACD- anterior chamber depth

Reefrences

1. Fieß A, Fauer A, Mildenberger E, et al. Refractive error, accommodation and lens opacification in adults born preterm and full-term: Results from the Gutenberg Prematurity Eye Study (GPES). *Acta ophthalmologica* 2022;100(7):e1439-e50. doi: 10.1111/aos.15116 [published Online First: 2022/03/18]
2. Fieß A, Dautzenberg K, Gißler S, et al. Prevalence of strabismus and risk factors in adults born preterm with and without retinopathy of prematurity: results from the Gutenberg Prematurity Eye study. *The British journal of ophthalmology* 2024 doi: 10.1136/bjo-2023-324698 [published Online First: 2024/03/20]
3. Fieß A, Nauen H, Mildenberger E, et al. Ocular geometry in adults born extremely, very and moderately preterm with and without retinopathy of prematurity: results from the Gutenberg Prematurity Eye Study. *The British journal of ophthalmology* 2023;107(8):1125-31. doi: 10.1136/bjophthalmol-2021-320907 [published Online First: 2022/03/12]
4. Fieß A, Hufschmidt-Merizian C, Gißler S, et al. Dry Eye Parameters and Lid Geometry in Adults Born Extremely, Very, and Moderately Preterm with and without ROP: Results from the Gutenberg Prematurity Eye Study. *Journal of clinical medicine* 2022;11(10) doi: 10.3390/jcm11102702 [published Online First: 2022/05/29]
5. Fieß A, Grabitz SD, Mildenberger E, et al. A lower birth weight percentile is associated with central corneal thickness thinning: Results from the Gutenberg Prematurity Eye Study (GPES). *Journal of optometry* 2023;16(2):143-50. doi: 10.1016/j.optom.2022.07.001 [published Online First: 2022/10/02]
6. Fieß A, Gißler S, Mildenberger E, et al. Anterior Chamber Angle in Adults Born Extremely, Very, and Moderately Preterm with and without Retinopathy of Prematurity-Results of the Gutenberg Prematurity Eye Study. *Children (Basel, Switzerland)* 2022;9(2) doi: 10.3390/children9020281 [published Online First: 2022/02/26]
7. Fieß A, Pfisterer A, Gißler S, et al. RETINAL THICKNESS AND FOVEAL HYPOPLASIA IN ADULTS BORN PRETERM WITH AND WITHOUT RETINOPATHY OF PREMATURITY: The Gutenberg Prematurity Eye Study. *Retina (Philadelphia, Pa)* 2022;42(9):1716-28. doi: 10.1097/iae.0000000000003501 [published Online First: 2022/08/23]
8. Fieß A, Zange M, Gißler S, et al. Foveal avascular zone in adults born preterm with and without retinopathy of prematurity - results from the Gutenberg Prematurity Eye Study. *Retina (Philadelphia, Pa)* 2024 doi: 10.1097/iae.0000000000004113 [published Online First: 2024/04/02]
9. Fieß A, Schäffler A, Mildenberger E, et al. Peripapillary Retinal Nerve Fiber Layer Thickness in Adults Born Extremely, Very, and Moderately Preterm With and Without Retinopathy of Prematurity: Results From the Gutenberg Prematurity Eye Study (GPES). *American journal of ophthalmology* 2022;244:88-97. doi: 10.1016/j.ajo.2022.07.019 [published Online First: 2022/08/07]
10. Fieß A, Gißler S, Mildenberger E, et al. Optic Nerve Head Morphology in Adults Born Extreme, Very, and Moderate Preterm With and Without Retinopathy of Prematurity: Results From the Gutenberg Prematurity Eye Study. *American journal of ophthalmology* 2022;239:212-22. doi: 10.1016/j.ajo.2022.03.005 [published Online First: 2022/03/16]

11. Fieß A, Berger LA, Riedl JC, et al. The role of preterm birth, retinopathy of prematurity and perinatal factors on corneal aberrations in adulthood: Results from the Gutenberg prematurity eye study. *Ophthalmic & physiological optics : the journal of the British College of Ophthalmic Opticians (Optometrists)* 2022;42(6):1379-89. doi: 10.1111/opo.13038 [published Online First: 2022/08/26]
12. Fieß A, Greven K, Mildenberger E, et al. Visual acuity, amblyopia, and vision-related quality of life in preterm adults with and without ROP: results from the Gutenberg prematurity eye study. *Eye (London, England)* 2023;37(9):1794-801. doi: 10.1038/s41433-022-02207-y [published Online First: 2022/09/16]
13. Fieß A, Volmering C, Gißler S, et al. Macular Curvature in Adults Born Preterm With and Without ROP: Results from the Gutenberg Prematurity Eye Study. *Investigative ophthalmology & visual science* 2024;65(3):39. doi: 10.1167/iovs.65.3.39 [published Online First: 2024/03/29]
14. Fieß A, Schuster AK, Nickels S, et al. Association of low birth weight with myopic refractive error and lower visual acuity in adulthood: results from the population-based Gutenberg Health Study (GHS). *The British journal of ophthalmology* 2019;103(1):99-105. doi: 10.1136/bjophthalmol-2017-311774 [published Online First: 2018/03/17]
15. Fieß A, Urschitz MS, Nagler M, et al. Association of birth weight with corneal aberrations in adulthood - Results from a population-based study. *Journal of optometry* 2023;16(1):42-52. doi: 10.1016/j.optom.2021.06.004 [published Online First: 2022/06/29]
16. Fieß A, Urschitz MS, Marx-Groß S, et al. Association of Birth Weight with Central and Peripheral Corneal Thickness in Adulthood-Results from the Population-Based German Gutenberg Health Study. *Children (Basel, Switzerland)* 2021;8(11) doi: 10.3390/children8111006 [published Online First: 2021/11/28]
17. Fieß A, Schuster AK, Nickels S, et al. Association of Low Birth Weight With Altered Corneal Geometry and Axial Length in Adulthood in the German Gutenberg Health Study. *JAMA ophthalmology* 2019;137(5):507-14. doi: 10.1001/jamaophthalmol.2018.7121
18. Fieß A, Wagner FM, Urschitz MS, et al. Association of Birth Weight With Foveolar Thickness in Adulthood: Results From a Population-Based Study. *Invest Ophthalmol Vis Sci* 2021;62(14):9. doi: 10.1167/iovs.62.14.9 [published Online First: 2021/11/11]
19. Fieß A, Nickels S, Urschitz MS, et al. Association of Birth Weight with Peripapillary Retinal Nerve Fiber Layer Thickness in Adulthood—Results from a Population-Based Study. *Investigative ophthalmology & visual science* 2020;61(8):4-4. doi: 10.1167/iovs.61.8.4
20. Fieß A, Lamparter J, Raum P, et al. Birth Weight and Diabetic Retinopathy: Results From the Population-Based Gutenberg Health Study (GHS). *Ophthalmic epidemiology* 2021;28(2):122-30. doi: 10.1080/09286586.2020.1800753 [published Online First: 2020/09/30]
21. Fieß A, Elbaz H, Korb CA, et al. Low Birth Weight Is Linked to Age-Related Macular Degeneration: Results From the Population-Based Gutenberg Health Study (GHS). *Invest Ophthalmol Vis Sci* 2019;60(14):4943-50. doi: 10.1167/iovs.19-27964 [published Online First: 2019/11/27]
22. Pétursdóttir D, Holmström G, Larsson E. Visual function is reduced in young adults formerly born prematurely: a population-based study. *The British journal of ophthalmology* 2020;104(4):541-46. doi: 10.1136/bjophthalmol-2019-314429 [published Online First: 2019/07/16]
23. Pétursdóttir D, Holmström G, Larsson E. Refraction and its development in young adults born prematurely and screened for retinopathy of prematurity. *Acta ophthalmologica* 2021 doi: 10.1111/aos.14766 [published Online First: 2021/02/03]
24. Pétursdóttir D, Holmström G, Larsson E. Strabismus, stereoacuity, accommodation and convergence in young adults born premature and screened for retinopathy of prematurity. *Acta ophthalmologica* 2022;100(3):e791-e97. doi: 10.1111/aos.14987 [published Online First: 2021/07/28]

25. Pétursdóttir D, Åkerblom H, Holmström G, et al. Central macular morphology and optic nerve fibre layer thickness in young adults born premature and screened for retinopathy of prematurity. *Acta ophthalmologica* 2024;102(4):391-400. doi: 10.1111/aos.15814 [published Online First: 2023/11/22]
26. Jain S, Sim PY, Beckmann J, et al. Functional Ophthalmic Factors Associated With Extreme Prematurity in Young Adults. *JAMA network open* 2022;5(1):e2145702. doi: 10.1001/jamanetworkopen.2021.45702 [published Online First: 2022/01/29]
27. Plotnikov D, Williams C, Guggenheim JA. Association between birth weight and refractive error in adulthood: a Mendelian randomisation study. *The British journal of ophthalmology* 2020;104(2):214-19. doi: 10.1136/bjophthalmol-2018-313640 [published Online First: 2019/05/18]
28. Lingham G, Mackey DA, Sanfilippo PG, et al. Influence of prenatal environment and birth parameters on amblyopia, strabismus, and anisometropia. *Journal of AAPOS : the official publication of the American Association for Pediatric Ophthalmology and Strabismus* 2020;24(2):74.e1-74.e7. doi: 10.1016/j.jaapos.2019.12.013 [published Online First: 2020/03/11]
29. Balasubramanian S, Beckmann J, Mehta H, et al. Relationship between retinal thickness profiles and visual outcomes in young adults born extremely preterm: The EPICure@19 Study. *Ophthalmology* 2019;126(1):107-12. doi: 10.1016/j.ophtha.2018.07.030 [published Online First: 2018/08/10]
30. Kistner A, Jacobson L, Jacobson SH, et al. Low gestational age associated with abnormal retinal vascularization and increased blood pressure in adult women. *Pediatric research* 2002;51(6):675-80. doi: 10.1203/00006450-200206000-00003 [published Online First: 2002/05/29]
31. Darlow BA, Elder MJ, Kimber B, et al. Vision in former very low birthweight young adults with and without retinopathy of prematurity compared with term born controls: the NZ 1986 VLBW follow-up study. *The British journal of ophthalmology* 2018;102(8):1041-46. doi: 10.1136/bjophthalmol-2017-311345 [published Online First: 2017/12/08]
32. Ghoraba HH, Ludwig CA, Moshfeghi DM. Biometric Variations in High Myopia Associated with Different Underlying Ocular and Genetic Conditions. *Ophthalmology science* 2023;3(1):100236. doi: 10.1016/j.xops.2022.100236 [published Online First: 2022/12/23]
33. Iwase S, Kaneko H, Fujioka C, et al. A long-term follow-up of patients with retinopathy of prematurity treated with photocoagulation and cryotherapy. *Nagoya journal of medical science* 2014;76(1-2):121-8. [published Online First: 2014/08/19]
34. Thanos A, Yonekawa Y, Todorich B, et al. Spectral-Domain Optical Coherence Tomography in Older Patients With History of Retinopathy of Prematurity. *Ophthalmic surgery, lasers & imaging retina* 2016;47(12):1086-94. doi: 10.3928/23258160-20161130-02 [published Online First: 2016/12/16]
35. Baker PS, Tasman W. Optical coherence tomography imaging of the fovea in retinopathy of prematurity. *Ophthalmic surgery, lasers & imaging : the official journal of the International Society for Imaging in the Eye* 2010;41(2):201-6. doi: 10.3928/15428877-20100303-08 [published Online First: 2010/03/24]
36. Uner OE, Rao P, Hubbard GB, 3rd. Reactivation of Retinopathy of Prematurity in Adults and Adolescents. *Ophthalmology Retina* 2020;4(7):720-27. doi: 10.1016/j.oret.2020.02.001 [published Online First: 2020/04/01]
37. Smith J, Shivitz I. Angle-closure glaucoma in adults with cicatricial retinopathy of prematurity. *Arch Ophthalmol* 1984;102(3):371-2. doi: 10.1001/archopht.1984.01040030289020 [published Online First: 1984/03/01]
38. Kaiser RS, Trese MT, Williams GA, et al. Adult retinopathy of prematurity: outcomes of rhegmatogenous retinal detachments and retinal tears. *Ophthalmology* 2001;108(9):1647-53. doi: 10.1016/s0161-6420(01)00660-1 [published Online First: 2001/09/06]
39. Kaiser RS, Fenton GL, Tasman W, et al. Adult retinopathy of prematurity: retinal complications from cataract surgery. *American journal of ophthalmology* 2008;145(4):729-35. doi: 10.1016/j.ajo.2007.11.007 [published Online First: 2008/01/30]

40. Hsu HT, Yu-Chuan Kang E, Blair MP, et al. Late Vitreoretinal Complications of Regressed Retinopathy of Prematurity: Retinal Break, Vitreous Hemorrhage, and Retinal Detachment. *Ophthalmology Retina* 2023;7(1):72-80. doi: 10.1016/j.oret.2022.07.005 [published Online First: 2022/07/18]
41. Hamad AE, Moinuddin O, Blair MP, et al. Late-Onset Retinal Findings and Complications in Untreated Retinopathy of Prematurity. *Ophthalmology Retina* 2020;4(6):602-12. doi: 10.1016/j.oret.2019.12.015 [published Online First: 2020/02/16]
42. Nilsson M, Hellström A, Jacobson L. Retinal Sequelae in Adults Treated With Cryotherapy for Retinopathy of Prematurity. *Invest Ophthalmol Vis Sci* 2016;57(9):Oct550-5. doi: 10.1167/iovs.15-18583 [published Online First: 2016/08/19]
43. Jacobson L, Vollmer B, Kistner A, et al. Severity of retinopathy of prematurity was associated with a higher risk of cerebral dysfunction in young adults born extremely preterm. *Acta paediatrica (Oslo, Norway : 1992)* 2021;110(2):528-36. doi: 10.1111/apa.15461 [published Online First: 2020/07/07]
44. Kulmala M, Jørgensen APM, Aakvik KAD, et al. Visual function in adults born preterm with very low birth weight-A two-country birth cohort study. *Acta ophthalmologica* 2024;102(1):49-57. doi: 10.1111/aos.15683 [published Online First: 2023/05/12]
45. Ingvaldsen SH, Jørgensen AP, Grøtting A, et al. Visual outcomes and their association with grey and white matter microstructure in adults born preterm with very low birth weight. *Scientific reports* 2024;14(1):2624. doi: 10.1038/s41598-024-52836-4 [published Online First: 2024/02/01]
46. Dirani M, Islam FM, Baird PN. The role of birth weight in myopia--the genes in myopia twin study. *Ophthalmic Res* 2009;41(3):154-9. doi: 10.1159/000209669 [published Online First: 2009/03/27]
